# Exogenous Ochronosis (EO): Skin lightening cream causing rare caviar-like lesion with banana-like pigments; review of literature and histological comparison with endogenous counterpart

**DOI:** 10.4322/acr.2020.197

**Published:** 2020-09-02

**Authors:** Amir Qorbani, Adnan Mubasher, George Peter Sarantopoulos, Scott Nelson, Maxwell Alexander Fung

**Affiliations:** 1 University of California, San Francisco (UCSF), Department of Pathology and Laboratory Medicine, San Francisco, CA, USA; 2 Icahn School of Medicine at Mount Sinai Beth Israel, Department of Diagnostic Pathology & Laboratory Medicine, New York, NY, USA; 3 University of California, Los Angeles (UCLA), Department of Pathology and Laboratory Medicine, Los Angeles, CA, USA; 4 University of California, Davis (UC Davis), Department of Pathology and Laboratory Medicine, Sacramento, CA, USA

**Keywords:** Ochronosis, Skin Care, Skin Cream, Skin Diseases, Skin Pigmentation

## Abstract

Ochronosis is a cutaneous disorder caused by the accumulation of phenols, either endogenously as homogentisic acid in patients with alkaptonuria (autosomal recessive disorder with deficiency of the enzyme homogentisic acid oxidase), or exogenously in patients using phenol products such as topical creams containing hydroquinone or the intramuscular application of antimalarial drugs. Exogenous ochronosis (EO) typically affects the face and was reported in patients with dark skin such as Black South Africans or Hispanics who use skin-lightening products containing hydroquinone for extended periods. Recently more cases have been reported worldwide even in patients with lighter skin tones, to include Eastern Indians, Asians, and Europeans. However, just 39 cases of EO have been reported in the US literature from 1983 to 2020. Here we present two cases; a 69 and a 45-year-old female who were seen for melasma, given hydroquinone 4% cream daily and tretinoin 0.05%. Both patients noticed brown spots on their cheeks, which progressively enlarged and darkened in color. The diagnosis of ochronosis was confirmed by characteristic histopathological features on the punch biopsy. Unfortunately, neither patient responded to multiple treatments (to include, tazarotene 0.1% gel and pimecrolimus ointment, topical corticosteroids, and avoidance of hydroquinone containing products). We also present a case of classic (endogenous) ochronosis in a patient with alkaptonuria to picture the histological similarities of these two entities. EO is an important clinical consideration because early diagnosis and treatment may offer the best outcome for this notoriously refractory clinical diagnosis.

## INTRODUCTION

Ochronosis an uncommon condition characterized by the accumulation of phenols, either endogenously as homogentisic acid in patients with alkaptonuria or exogenously in patients using topical products containing phenols. It was initially described in 1865 by Rudolf Virchow, based on the deposition of microscopic light brownish-yellow (ochre) pigments in a patient with endogenous ochronosis.^1^ ^,^ ^2^ Endogenous ochronosis occurs as a manifestation of alkaptonuria, a rare autosomal recessive condition resulting in loss of homogentisic acid oxidase. Homogentisic acid oxidase is involved in the metabolism of tyrosine and phenylalanine, and its absence leads to the accumulation of homogentisic acid (HGA) in the body, which causes the dark blue discoloration of cartilaginous tissue such as ear cartilage or articular surfaces. According to Laymon,^2^ exogenous ochronosis (EO) was first coined in 1906 by L. Pick, which is characterized by a bluish-gray patches with a coarse texture and “caviar-like” papules. EO typically affects face and neck and mainly seen in patients with dark skin. It was reported in Black South Africans^3^ or Hispanics,^4^ who used certain skin-lightening products containing phenol substances for a long time.^5^ Many drugs have been implicated in its etiology, including benzene, quinone, antimalarials drugs, and mercury derivatives. EO is more common in dark-skin individuals due to the application of skin lightening/bleaching products containing hydroquinone. To our knowledge, there are 39 cases in English literature, reported in the United States. We herein describe two cases of EO. For comparison, we also present a case of endogenous ochronosis involving the knee articular surface.

### Case Reports

**Figure 1:**
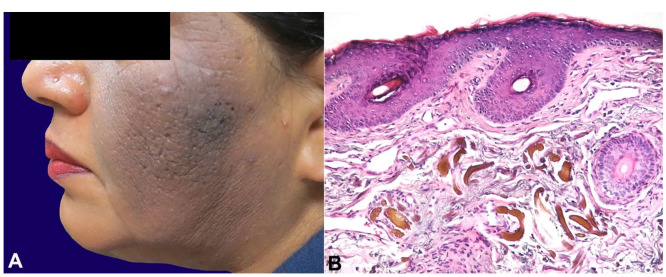
**A –** Exogenous ochronosis. Case#1; 58-year-old woman with melasma, treated with HQ 4% and tretinoin, progressed to itchy, tender, and more darken macules; **B –** Photomicrography of the skin biopsy of skin case #1, showing elongated yellow-brown deposits (“banana-like”) of acellular material in the superficial dermis with associated solar elastosis (H&E, 200X magnification).

**Case#1:** A 58-year-old female with a history of melasma was treated with hydroquinone 4% cream daily and tretinoin cream with some improvement. After a year, she noticed a red, scaly patch on her left cheek that progressively grew and darkened in color. The patches became gray-black, itchy, and tender (Figure 1A). Due to the history of long-term use of hydroquinone, and the asymmetrical presentation of the facial lesion, the clinical diagnosis of exogenous ochronosis was favored. Histological examination showed yellow-brown sharply defined irregularly shaped pigment granules in the upper dermis, diagnostic of exogenous ochronosis (Figure 1B). She discontinued the hydroquinone and tretinoin, and started desonide 0.5% lotion with sun protection. After a month, lesional inflammation was decreased, but the hyperpigmentation and a mild thickening persisted. She continued hydrocortisone cream and sun protection creams with minimal improvement in her symptoms.

**Figure 2:**
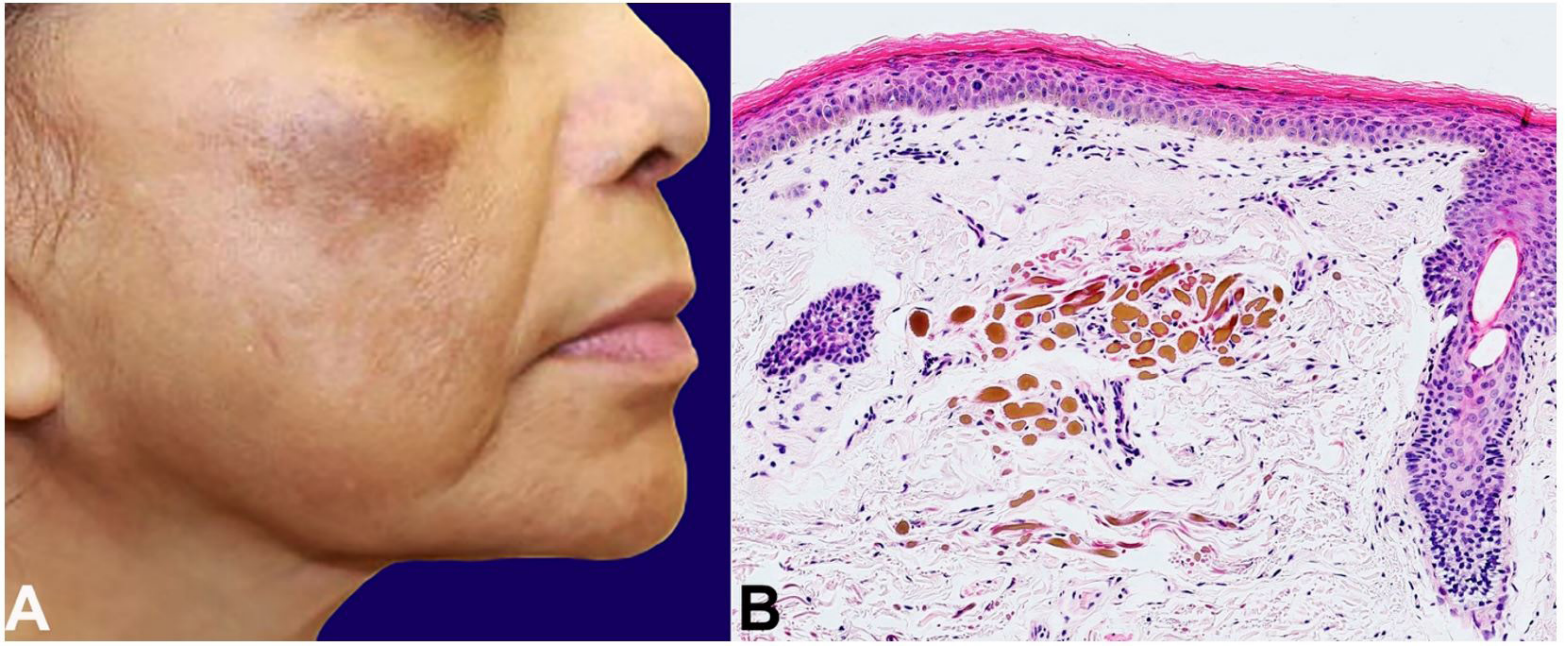
**A –** Exogenous ochronosis. Case #2, 68-year-old female with hyperpigmented macules which did not respond to HQ 4% and tretinoin 0.05%. The macules gradually got darker and erythematous with surrounding hypopigmentation; **B –** Photomicrography of the skin biopsy of skin case #2, showing banana-like bodies (ochronotic bodies) in the superficial dermis (H&E, 100X magnification).

**Case #2:** A 68-year-old female presented with clinically diagnosed chronic melasma characterized by dark brown hyperpigmented macules coalescing into patches on both cheeks. She did not respond to tretinoin 0.05% cream and hydroquinone 4% cream daily for one year. The cheek macules became erythematous and darker with hypopigmentation of the surrounding area (Figure 2A). Histological examination showed yellow-brown dermal deposits with “banana bodies,” diagnostic of ochronosis (Figure 2B). She started tazarotene 0.1% gel with pimecrolimus cream and sun protection. Unfortunately, one year of follow up showed minimal to no improvement.

**Figure 3:**
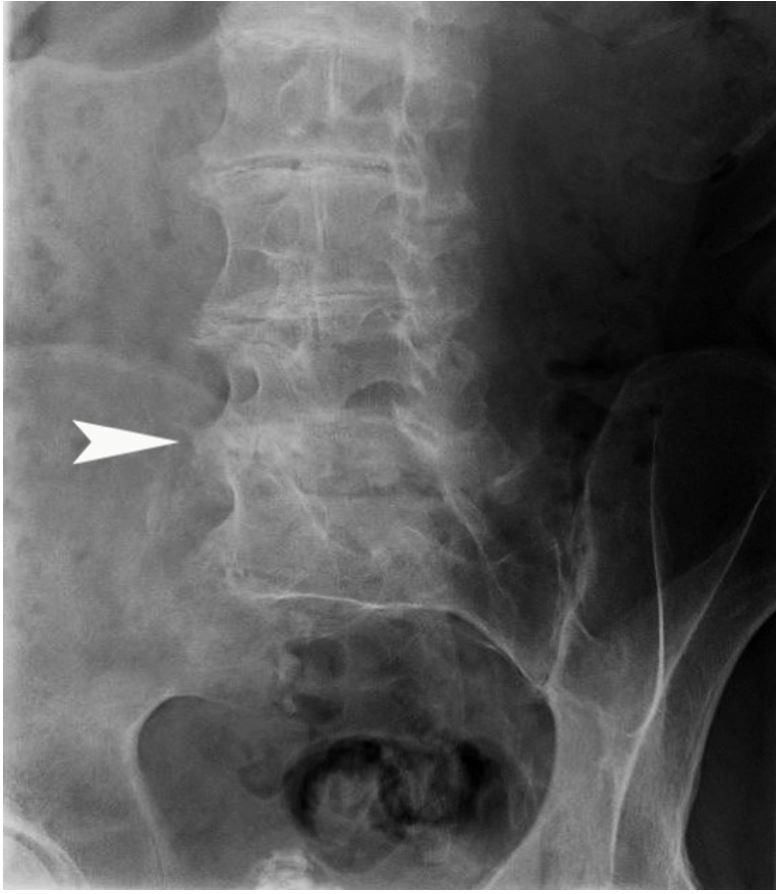
Radiographic findings in alkaptonuria. X-Ray radiography of lumbar spine showing extensive lumbar disc degeneration with disproportionately minor osteophytes and calcifications.

**Case #3:** A 63-year-old male with a history of knee, hip, and shoulder osteoarthritis and lumbar degenerative disc disease presented for management of severe multifocal osteoarthritis. Pigmentation of both ears was noted on physical examination, which was reportedly congenital. Radiological findings showed severe and extensive lumbar disc and facet joint degeneration with disproportionately minor osteophytes and calcification of numerous discs, that favored ochronosis with ochronosis-associated arthropathy (Figure 3).

**Figure 4:**
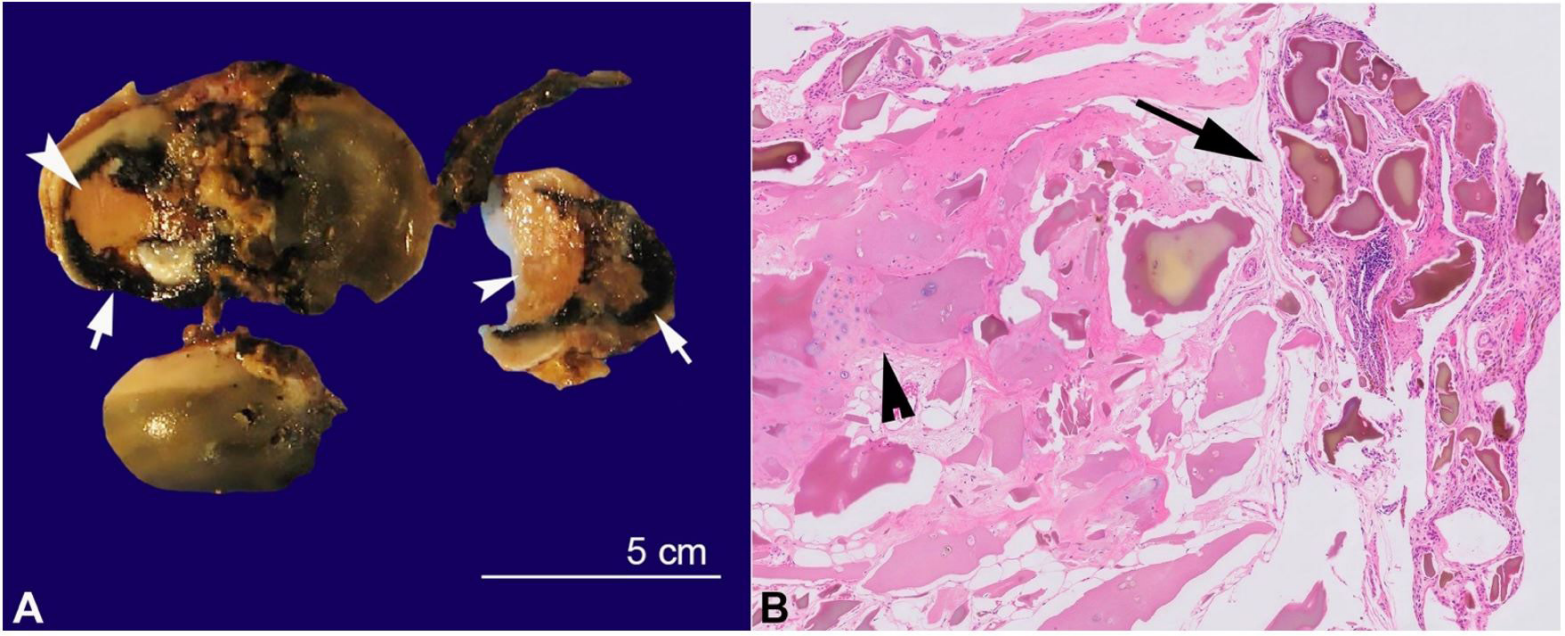
**A –** Alkaptonuria. Gross examination of total knee arthroplasty showing unusual dark articular surface (head) with eburnation (arrow head); **B –** Photomicrograph of the synovium of the total arthroplasty in patient with Alkaptonuria, showing ochronotic bodies in the synovium (arrow) and articular surface (arrow head). (H&E stain, 40X magnification).

Subsequent urine organic acid analysis revealed elevated homogentisic acid, confirming the diagnosis of alkaptonuria and endogenous ochronosis. He underwent bilateral total knee arthroplasty. Gross examination showed dark black articular surfaces with eburnation (Figure 4A). Histological examination showed extensive ochronotic pigment deposition in fibrocollagenous and synovial tissue, diagnostic of endogenous ochronosis (Figure 4B).

## DISCUSSION

Ochrone means bright yellow in Latin. The term ochronosis was first coined by Rudolf Virchow (1821-1902) in 1865, describing the strange dark lesions in an elderly man on autopsy, which on H&E were attributed to the presence of yellowish-brown pigments on light microscopic examination.

Two types of ochronosis have been described, exogenous and endogenous. Endogenous ochronosis is caused by alkaptonuria, an autosomal recessive disorder in which the absence of homogentisic acid oxidase leads to accumulation of homogentisic acid (HGA) in the body. HGA is soluble in water and circulates in the blood. It is polymerized into a yellow-green pigment in the susceptible connective tissue. It binds irreversibly with the developing collagen fibers and imparts a dark color to the affected tissue. Certain tissues are more susceptible to the accumulation of HGA, including skin and especially articular cartilage of joints. The most common areas of skin involvement in alkaptonuria are the nasal tip, sclera of the eyes, cheeks, and the pinnae of ears. These patients excrete HGA in urine, which is oxidized by ambient oxygen to benzoquinone acetate, turning the urine dark black. The diagnosis of alkaptonuria is usually made in the third or fourth decade of life.

Exogenous ochronosis was described later by Pick^2^ and then in further detail by Beddard and Plumtre^6^ describing phenol (carbolic oil) as one of the causative agents for EO. Exogenous or pseudo-ochronosis is an infrequent dermatosis and is essentially the same entity as endogenous ochronosis but without the systemic complications. As the name implies, it is caused by the exogenous application of certain products leading up to the accumulation of polymerized HGA in collagen-containing tissue, including the dermis, as a bright yellow pigment (ochre). Clinically it presents as blue-black macules and/or minute papules in the malar area, cheeks, forehead, and neck as most of the cosmetic products are applied in these areas. Hydroquinone,^7^ other phenolic compounds (especially phenol carbolic acid),^6^ ^,^ ^8^ systemic antimalarial agents such as quinine injections,^9^ oral antimalarial drugs, benzene substances, and picric acid, to name a few chemicals that have been associated with causing exogenous ochronosis. Some authors believe exogenous ochronosis could be a granulomatous disorder, as it was seen in patients with sarcoidosis and considering the fact that hydroquinone products can exacerbate the granulomatous reactions in patients.^10^

The exact pathogenesis of exogenous ochronosis is not clear. Multiple mechanisms have been proposed to explain EO, including i) high concentration of hydroquinone stimulates tyrosinase leading to increased pigmentation of the affected tissue; ii) local inhibition of activation of homogentisic acid oxidase leading to accumulation of homogentisic acid and sunlight activation of ochronotic pigments.^11^ Hull and Procter^12^ suggested a causative role of melanocytes, as they reported melanocyte-free areas of an ochronotic patient with vitiligo were not hyperpigmented.^13^

In humans, the phenols form complexes such as hydroquinone are excreted after conjugation with glucuronic acid or sulfur. These metabolic pathways can only handle a small amount to process and excrete. In the situation of excess absorption of these substances, the excretory pathways are overwhelmed, leading to the accumulation of these substances in the tissues. It has been observed that the tissues where oxidation pathways are active in the extracellular matrix, hydroquinone, and phenols are oxidatively polymerized and stored as a dark amorphous solid pigment. This pigment is then taken up by the developing fibers and remains in the extracellular matrix of these tissues. This pigment accumulation has not been shown to affect the functionality of the tissue. The prolonged accumulation of polymerized homogentisic acid stimulates melanin production, thus, leading to a blue-black hue/ting to the tissue.^5^ The dark-blue or grayish-blue pigment is visible in the skin when this process takes place in the dermis, especially the dermis of the face because of excessive and prolonged use of hydroquinone containing products and depends on the depth of pigment accumulation. Clinically, it can manifest as macules and papules obliterating the follicular orifices with accentuation of the normal pseudo network of the skin.

A recent study at Boston University on 11 patients^14^ showed that topically applied hydroquinone (HQ) affects both elastic fibers and melanin production; HQ inhibits homogentisate 1,2dioxygenase (HGD) leading to accumulation HGA, which will be engulfed by fibroblasts that causes aberrant elastic fiber production. HQ also inhibits tyrosinase, which leads to increase immaturity and apoptosis of melanosomes, result in decrease photoprotection and deeper penetration of UV radiation and increase solar elastosis. These two effects lead to HGA and solar elastotic fibers, which eventually develop ochronotic bodies in elastosis.

By far, the most notorious offender is hydroquinone products. It is widely used as a bleaching agent to decrease the pigmentation of the skin. Hydroquinone is a mild local anesthetic and acts by reducing the number of melanocytes, decreasing the production of melanin by the remaining melanocytes, and bleaching the melanin pigment in the skin.^15^ It has been in use for the treatment of multiple hyper-pigmented conditions and is considered an excellent agent for conditions such as melasma.^16^ It was estimated that more than 10 million tubes containing hydroquinone containing compounds or related known ochronotic products are sold over the counter each year in the United States.^17^ On August 29, 2006, the U.S. Food and Drug Administration (FDA) proposed a ban on over-the-counter sales of cosmetic products containing hydroquinone as skin-bleaching (lightening) ingredient.

Histologically, endogenous, and exogenous ochronosis have identical features. Curvilinear yellow-green elastic fibers and banana-shaped amorphous material are seen in the lesional dermis. The overlying epidermis is usually normal, but pseudoepitheliomatous hyperplasia can occur.^5^ ^,^ ^10^

In long-standing disease, ochronotic fibers may degenerate to form colloid milium. Prominent inflammatory changes may include multinucleated giant cells, epithelioid histiocytes, and plasma cells.^18^ Dermoscopy, reflectance confocal microscopy, microscopy with UV surface excitation, or other techniques could also be a helpful tool in the diagnosis of ochronosis.^19^ ^,^ ^20^

The clinical manifestations have been described in different stages by Hardwick et al.^21^ in their epidemiological study:

Grade I – Faint macular sooty pigmentation
Grade II – Distinct macular stippling/small caviar-like papules^11^
Grade III – Dark deposits and papules
Grade IV – Colloid milia (1 mm and greater)
Grade V – Keloid-like nodules and cysts

A review of the English-speaking literature was performed using the PubMed database by searching the terms “ochronosis” and “Exogenous Ochronosis”. Incidence of exogenous ochronosis (EO) has been extremely low in the United States (US), and only 39 cases have been described in the English literature since 1983 to 2020 (Table 1).

**Table 1:** Exogenous Ochronosis Reported in the US from 1983 to 2020.

**#**	**Year**	**Author**	**PMID**	**Age**	**Sex**	**Ethnicity**	**Dosage Used**	**Duration**
1	1983	Cullison	6863651	58	F	Black	2% HQ	2.5 years
2	1985	Hoshaw	3966811	75	F	Black	2% HQ	2 years
3	1985	Hoshaw	3966811	49	F	Black	OTC skin lightening cream	2 months
4	1985	Penneys	4037813	NA	NA	NA	NA	NA
5	1987	Connor	3800410	72	F	Black	Skin-lightening cream	Many years
6	1988	Lang	3192777	74	F	White	HQ and Mercury cream	Many years
7	1988	Lawrence	3372785	62	F	Black	1% HQ	2-3 years
8	1988	Lawrence	3372785	46	F	Black	1% HQ	NA
9	1988	Fisher	3203531	47	F	Black	4% HQ	18 months
10	1990	Howard	2311433	36	F	Hispanic	2% HQ	4 months
11	1990	Diven	2246407	53	F	Black	2% HQ	2-3 months
12	1991	Jordaan	1928626	56	M	Black	6.5-7.5% HQ	Many years
13	1991	Jordaan	1928626	39	F	Black	Skin-lightening cream	5 years
14	1992	Martin	1603931	44	F	Black	2% HQ	3-4 years
15	1992	Martin	1603931	56	F	Black	OTC skin-lightening creams	30 years
16	1993	Snider	8463477	59	F	Black	2-4% HQ	Many years
17	1995	Jacyk	7695007	49	F	Black	NA	NA
18	1995	Jacyk	7695007	50	F	Black	NA	NA
19	1995	Jacyk	7695007	70	F	Black	NA	NA
20	1995	Jacyk	7695007	40	F	Black	NA	NA
21	1995	Jacyk	7695007	49	F	Black	NA	NA
22	1995	Jacyk	7695007	43	F	Black	NA	NA
23	2000	Kramer	10767690	50	F	Hispanic	2% HQ	30 years
24	2001	Bowman	11534909	75	F	Black	2% HQ	up to 1 year
25	2004	Bellew	15056151	47	F	Black	NA	Many months
26	2004	Bellew	15056151	46	M	Native American	NA	1 year
27	2006	Huerta	16468299	70	F	NA	2% HQ	6 years
28	2008	Merola	19061605	55	F	NA	NA	Many years
29-39	2019	Ho	31725487	5^th^-7^th^	F	Black	NA	NA

NA: Data is not available, F: Female, M: Male, OTC: Over the counter, HQ: hydroquinone.

One possible explanation can be attributed to EO being under reported or underdiagnosed. Hyperpigmentation and melasma commonly affect the female population of the African, Hispanic and Asian descends. In certain Asian communities, the incidence of hyperpigmentation is estimated to be around 40%.^16^ As a result of demographic and cultural shifts, it is reasonable to believe that the use of bleaching and whitening compounds, including hydroquinone products, may increase, leading to the comparatively more frequent encounter of exogenous ochronosis in the US.

Treatment of OE has been unsatisfactory with the achievement of sub-optimal results. Cryotherapy, tretinoin, and topical acetic acid have been used without dramatic success. However, promising results have been reported using Q-switched alexandrite laser,^22^ Q-switched ruby laser,^23^ CO2 laser and dermabrasion.^24^ Nevertheless, EO remains a difficult condition to treat, emphasizing the importance of early detection.

A review of the literature for EO in the US revealed that most of the cases were the result of 1-2% HQ creams with only a handful reporting use of the 3-4% hydroquinone. Thus, low concentrations of these products applied for a longer period, or increased frequency can also result in EO.^25^ ^-^ ^27^ Furthermore, EO is a psychologically debilitating condition that impairs quality of life. It is of utmost significance to distinguish EO from melasma, Ota nevus, drug-induced hyperpigmentation, post-inflammatory pigmentation, and dermatosis papulosa nigra, in part because of the risk of being prescribed the very causative agent of the disease, leading to further worsening of the condition.
